# Obstructive uropathy associated with rheumatoid arthritis successfully treated with steroids and immunosuppressive therapy: A case report

**DOI:** 10.1097/MD.0000000000018415

**Published:** 2019-12-16

**Authors:** Ru-Xuan Chen, Siyan “Stewart” Cao, Li-Dan Zhao, Hua-Xia Yang

**Affiliations:** a Department of Rheumatology and Clinical Immunology, Peking Union Medical College Hospital, Clinical Immunology Center, Medical Epigenetics Research Center, Chinese Academy of Medical Sciences and Peking Union Medical College, Beijing, China; b Department of Medicine, University of California, San Francisco, California, USA.

**Keywords:** corticosteroids, hematuria, immunosuppressive therapy, rheumatoid arthritis, urinary obstruction

## Abstract

**Rationale:**

Urinary obstruction are relatively rare complications of autoimmune diseases including systemic lupus erythematosus and systemic vasculitis. It has never been reported in rheumatoid arthritis (RA).

**Patient concerns:**

We report a case of a female patient with seropositive RA who presented with gross hematuria associated with worsening joint symptoms, found to have acute kidney injury (AKI), bilateral hydronephrosis with bilateral renal pelvis, and ureteral wall thickening. Uroscopy with biopsy demonstrated inflammation without evidence of malignancy.

**Diagnoses:**

Rheumatoid arthritis related inflammation and obstruction of the urinary tract.

**Interventions:**

Prednisone 50 mg daily (tapering began 1 month later), iguratimod 50 mg daily, and leflunomide 20 mg daily were prescribed.

**Outcomes:**

The patient responded well to steroids and immunosuppressive therapy with complete resolution of hematuria, renal injury, and hydronephrosis.

**Lessons:**

Our case showed that RA might cause local inflammation involving the urinary tract which leads to obstruction and AKI.

## 1 Introduction

Urinary manifestations are relatively rare complications of autoimmune diseases.^[1–7]^ Lupus cystitis and hydronephrosis have been reported in systemic lupus erythematosus (SLE) patients.^[1–2]^ Interstitial cystitis was reported in Sjögren syndrome.^[3]^ And systemic vasculitis can present as obstructive uropathy including urethral stricture or bladder outlet obstruction.^[4–7]^ Obstructive uropathy associated with rheumatoid arthritis (RA) has not been reported in English literature before based on our knowledge. In this study, we report the first case of gross hematuria and renal injury in the settings of urinary tract wall thickening and bilateral hydronephrosis in a female RA patient, who responded well to steroids and immunosuppressive therapy.

## 2 Case report

The patient is a 52 year-old Chinese woman, previously healthy, who first developed joint pain and morning stiffness affecting bilateral hands, wrists, elbows, shoulders, knees, and temporomandibular joints with elevated rheumatoid factor and anti-cyclic citrullinated peptide antibody, and was diagnosed of RA at a local hospital in 2013. The patient was started on methotrexate 10 mg weekly, leflunomide 10 mg daily with gradual improvement of her joint symptoms. Methotrexate was discontinued in March 2014 due to suspected pulmonary side effect. The patient experienced worsening joint symptoms in March 2015. Prednisone 10 mg daily and iguratimod 25 mg twice daily were added to leflunomide 10 mg daily with subsequent improvement of symptoms. Prednisone was tapered off in February 2016 with continuation of iguratimod 25 mg daily and leflunomide 10 mg daily.

In July 2016 the patient developed sudden-onset right flank cramping pain, dysuria, and intermittent gross hematuria with blood clots; no urinary urgency/frequency, fever/chills, nausea/vomiting, or diarrhea. Workups at a local hospital showed elevated red blood cells and white blood cells on urinalysis, negative urinary bacterial culture, right-sided hydronephrosis, and dilated right ureter on renal ultrasound. The patient was prescribed a course of levofloxacin empirically for urinary tract infection with resolution of flank pain and dysuria but continued to have intermittent gross hematuria. In February 2017 the patient started having more frequent episodes of gross hematuria in the settings of worsening pain affecting multiple joints which were similar to her previous RA exacerbation. She had no flank pain, dysuria, urinary urgency/frequency, or fever/chills. Iguratimod was increased to 25 mg twice daily and continued on leflunomide 10 mg daily, which alleviated joint symptoms but failed to relieve gross hematuria. The patient then presented to our hospital for further workup and treatment.

**Figure 1:**
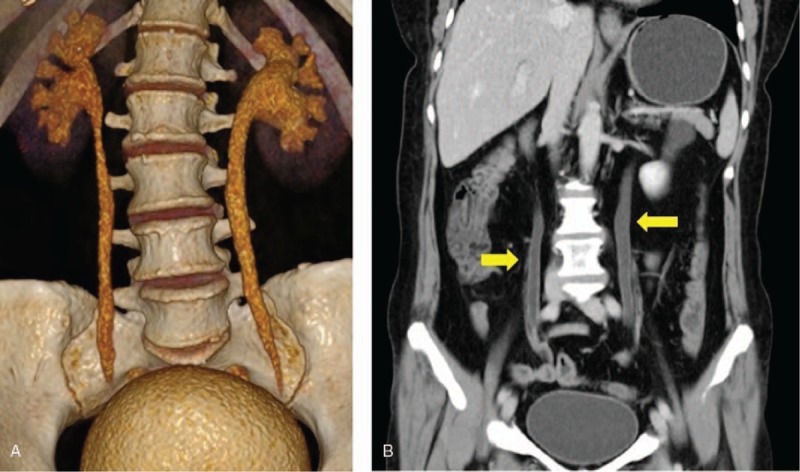
CT urography shows bilateral hydronephrosis, wall thickening of bilateral renal pelvis, and whole length of bilateral ureters with abnormal enhancement.

**Figure 2:**
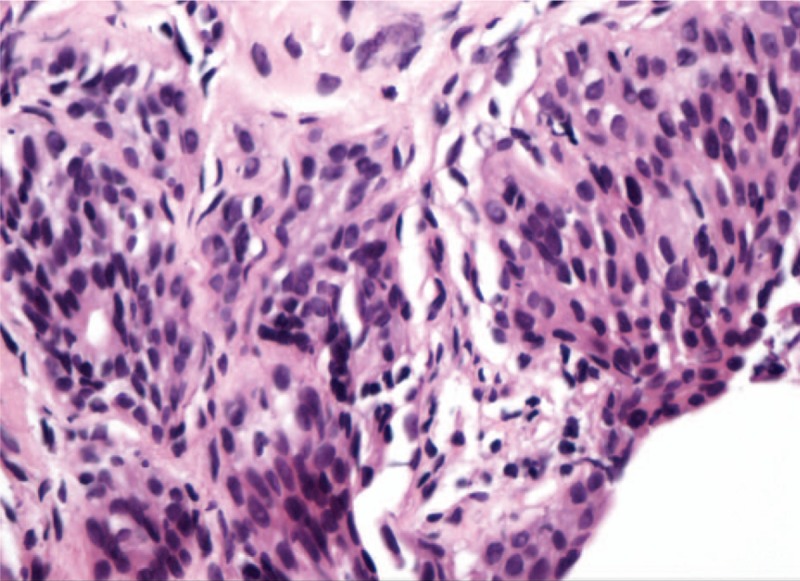
Pathology of vesicular trigone mucosal tissue consistent with chronic inflammation.

The patient endorsed active gross hematuria without flank or abdominal pain, dysuria, urinary urgency/frequency, fever/chills, nausea/vomiting, diarrhea, numbness, or weakness. Vital signs were stable upon admission. Physical examinations showed tenderness and swelling of left 2nd proximal interphalangeal (PIP) joint and right 4th PIP joint, swelling of right 3rd metacarpophalangeal joint, swelling of the left knee. She denied lymphadenopathy, lower extremity edema, abdominal tenderness, or distension; no pulmonary, cardiac, and neurologic examinations unremarkable. Lab tests including complete blood counts, liver function tests, coagulation tests were unremarkable. Urinalysis showed large number of white blood cells, red blood cells with normal morphology. Multiple urinary cultures for bacterial or fungal infection and urinary cytology for malignancy were negative. Creatinine was elevated at 89 μmol/L (baseline normal; normal reference range: 45–84 μmol/L); erythrocyte sedimentation rate was 67 mm/h, C-reactive protein was 8.64 mg/L; serum complements, immunoglobulin, IgG4 were within normal limit; rheumatoid factor was 282.8IU/ml, anti-cyclic citrullinated peptide antibody was 293U/ml; anti-nuclear antibodies and anti-neutrophil cytoplasmic antibodies were negative. Urine acid-fast staining and Mycobacterium tuberculosis DNA PCR were negative. Serum protein electrophoresis, serum, and urine immunofixation electrophoresis, urine free light chain analyses were all negative. CT urography showed bilateral hydronephrosis as well as wall thickening of bilateral renal pelvis and whole length of bilateral ureters with abnormal enhancement (Fig. 1). Obstructive uropathy was suggested. To rule out malignancy, cystoscopy was performed and demonstrated no definitive space-occupying lesion. Pathology of vesicular trigone mucosal tissue was consistent with chronic inflammation (Fig. 2). Considering that the patient's urinary tract wall thickening, inflammation, and gross hematuria could be related to RA, the patient was started on prednisone 50 mg daily (tapering began 1 month later), iguratimod 50 mg daily, and leflunomide 20 mg daily. At 3-month follow-up, the patient's hematuria resolved on urinalysis with serum creatinine back to normal. Repeated renal ultrasound at a local hospital 1.5 year later demonstrated complete resolution of bilateral hydronephrosis.

## 3 Discussion

We report a case of RA presenting with hematuria, bilateral hydronephrosis, and post-renal AKI. RA-associated obstructive uropathy was previously unknown and this is the first case reported in English literature to the best of our knowledge. However, several other autoimmune diseases have been reported to affect the urinary system. Lupus cystitis and hydronephrosis are rare complications of SLE. Interestingly, the urinary manifestations were associated with gastrointestinal symptoms in these patients, although the underlying mechanisms are unclear. Most SLE patients with lupus cystitis and/or hydronephrosis responded to corticosteroids with or without cyclophosphamide; while delayed diagnosis and treatment were associated with irreversible obstructive uropathy and permanent renal insufficiency in previous studies.^[1,2]^ Interstitial cystitis was reported in a patient with Sjögren syndrome and successfully treated with corticosteroids and tacrolimus.^[3]^ Vasculitis can rarely cause urinary obstruction. Granulomatosis with polyangiitis commonly affects the kidneys, although urologic involvement is extremely rare. Two cases were reported in English literature that urethral obstruction and acute urinary retention developed in patients with granulomatosis with polyangiitis. In the more recent case the patient was treated with high dose steroids and methotrexate.^[4,5]^ Similarly, ureteral stenosis was reported in a female pediatric patient with necrotizing vasculitis.^[6]^ Polyarteritis nodosa of the urinary tract is also very rare. In a case report, a 27-year-old male presented with chronic urinary obstruction found to have hydronephrosis and a bladder neck mass with medium-sized necrotising vasculitis on pathology. The patient met the diagnostic criteria for polyarteritis nodosa and responded to the combination of intravenous cyclophosphamide and oral corticosteroids.^[7]^

In our study, the patient presented with gross hematuria in the settings of RA exacerbation and found to have AKI, bilateral hydronephrosis, and wall thickening of bilateral renal pelvis and bilateral ureters. Pathology of vesicular trigone mucosa was consistent with chronic inflammation, which did not resolve after antibiotic treatment and was more likely to represent the urinary manifestation of RA. Based on this hypothesis, the patient was started on oral steroids with increased dosing of immunosuppressants, which led to complete resolution of hematuria, renal injury, and hydronephrosis. This patient's response was similar to previous reported cases of different autoimmune conditions as discussed above. Fortunately, the patient's delayed diagnosis and treatment with steroids did not cause permanent obstructive uropathy and/or renal impairment.

Finally, urinary tract infection and urologic malignancy must be ruled out in any patient with gross hematuria and obstructive uropathy. In this case, the patient completed a course of antibiotics at an outside hospital which resolved her flank pain and dysuria but failed to alleviate her hematuria. It is possible that the patient might have had a urinary tract infection in the settings of urinary obstruction, although urinary tract infection alone could not explain her persistent hematuria status post antibiotic therapy. Prolonged immunosuppressive therapy associates with increased risks of malignancy. Cystoscopy showed no definitive space-occupying lesion with bladder pathology negative for cancer in this case.

## 4 Conclusion

RA is a systemic autoimmune condition that can affect multiple organ systems. This is the first case that RA caused urinary tract wall inflammation and thickening leading to hydronephrosis, hematuria, and post-renal AKI, which completely resolved with steroids and immunosuppressive therapy. Further studies should explore the underlying mechanisms of this complication and formulate early intervention to avoid possible irreversible obstructive uropathy and permanent renal injury.

## Author contributions

**Conceptualization:** Ru-Xuan Chen, Li-Dan Zhao, Hua-Xia Yang.

**Data curation:** Ru-Xuan Chen, Siyan Stewart Cao.

**Investigation:** Ru-Xuan Chen, Siyan Stewart Cao.

**Methodology:** Ru-Xuan Chen.

**Project administration:** Ru-Xuan Chen.

**Supervision:** Hua-Xia Yang.

**Writing – original draft:** Ru-Xuan Chen, Siyan Stewart Cao.

**Writing – review & editing:** Ru-Xuan Chen, Siyan Stewart Cao, Li-Dan Zhao, Hua-Xia Yang.

Ru-Xuan Chen orcid: 0000-0002-8808-5322.

Hua-Xia Yang orcid: 0000-0001-6811-0255.
